# High release of hexavalent chromium into artificial sweat in a case of leather shoe–induced contact dermatitis

**DOI:** 10.1111/cod.13425

**Published:** 2019-11-06

**Authors:** Yolanda S. Hedberg, Zheng Wei, Mihály Matura

**Affiliations:** ^1^ KTH Royal Institute of Technology, School of Engineering Sciences in Chemistry, Biotechnology, and Health, Department of Chemistry Division of Surface and Corrosion Science Stockholm Sweden; ^2^ Unit of Occupational and Environmental Dermatology Centre for Occupational and Environmental Medicine, Stockholm County Council Stockholm Sweden; ^3^ Unit of Dermatology Skaraborgs Hospital Skövde Skövde Sweden

Cases of allergic contact dermatitis due to chromium caused by chromium‐tanned leather articles such as shoes and gloves are well‐known.^1^, ^2^ In the European Union, hexavalent chromium in new leather articles on the market is restricted to 3 mg/kg since 2015.^3^ A recent use‐test study showed that also trivalent chromium, the dominating form of chromium released from new leather articles,^4^ can elicit allergic reactions in chromium‐allergic individuals.^5^ For leather articles that are used for a prolonged period, data on the released chromium form are scarce, but one long‐term study indicates that the fraction of hexavalent chromium increases with duration of use.^6^

## CASE REPORT

A 36‐year‐old male patient working as a boat carpenter with a previous history of psoriasis and smoking, but no other known skin disease or allergy, presented with severe eczema showing papules and vesicles at both hands and feet with unknown cause for about half a year. Due to exposure to various occupational allergens an extensive patch test investigation (Swedish baseline series, extended series, rubber complementary series, epoxy series, and formaldehyde releaser series) was conducted. Patch testing showed a strong (++) reaction to potassium dichromate 0.5% pet. and a positive (+) reaction to cobalt(II) chloride hexahydrate 1% pet.; no other occupational allergens were revealed. The patient stopped wearing leather shoes and gloves, and the eczema improved significantly already after some days. About 80% of the inflammation was reduced within 14 days. The patient described that he was wearing sailing leather shoes without socks during summer and that his work shoes and some gloves were made of leather. A diphenylcarbazide spot test showed a positive result for hexavalent chromium for one pair of the shoes and a doubtful result for the other shoes, and therefore, chemical analysis was conducted for all shoes.

## CHEMICAL ANALYSIS OF SHOES

Chemical analysis of different parts of the shoes were conducted as in References 5 and 6. In short, six pieces of each type were preconditioned in a desiccator with less than 10% humidity and at room temperature for at least 1 week. Three pieces were then incubated, along with one vessel without leather as negative control, for 3 hours in phosphate buffer (22.8 g/L K_2_HPO_4_·3H_2_O, pH 8.0) at 25°C, as required in the standard test for restriction of hexavalent chromium in leather.^7^ Another three pieces and a negative control for each type were incubated at 30°C in artificial sweat for 24 hours (5 g/L NaCl, 1 g/L urea, 1 g/L lactic acid, pH 6.5). The leather pieces were then separated from the solution, and the solution was analyzed for total chromium and cobalt by means of atomic absorption spectroscopy (detection limit of 0.1 mg/L or 5 mg/kg), and for hexavalent chromium by means of UV–vis spectroscopy (detection limit of 0.02‐0.06 mg/L or 1‐3 mg/kg), as described previously.^4^, ^6^

**Figure 1:**
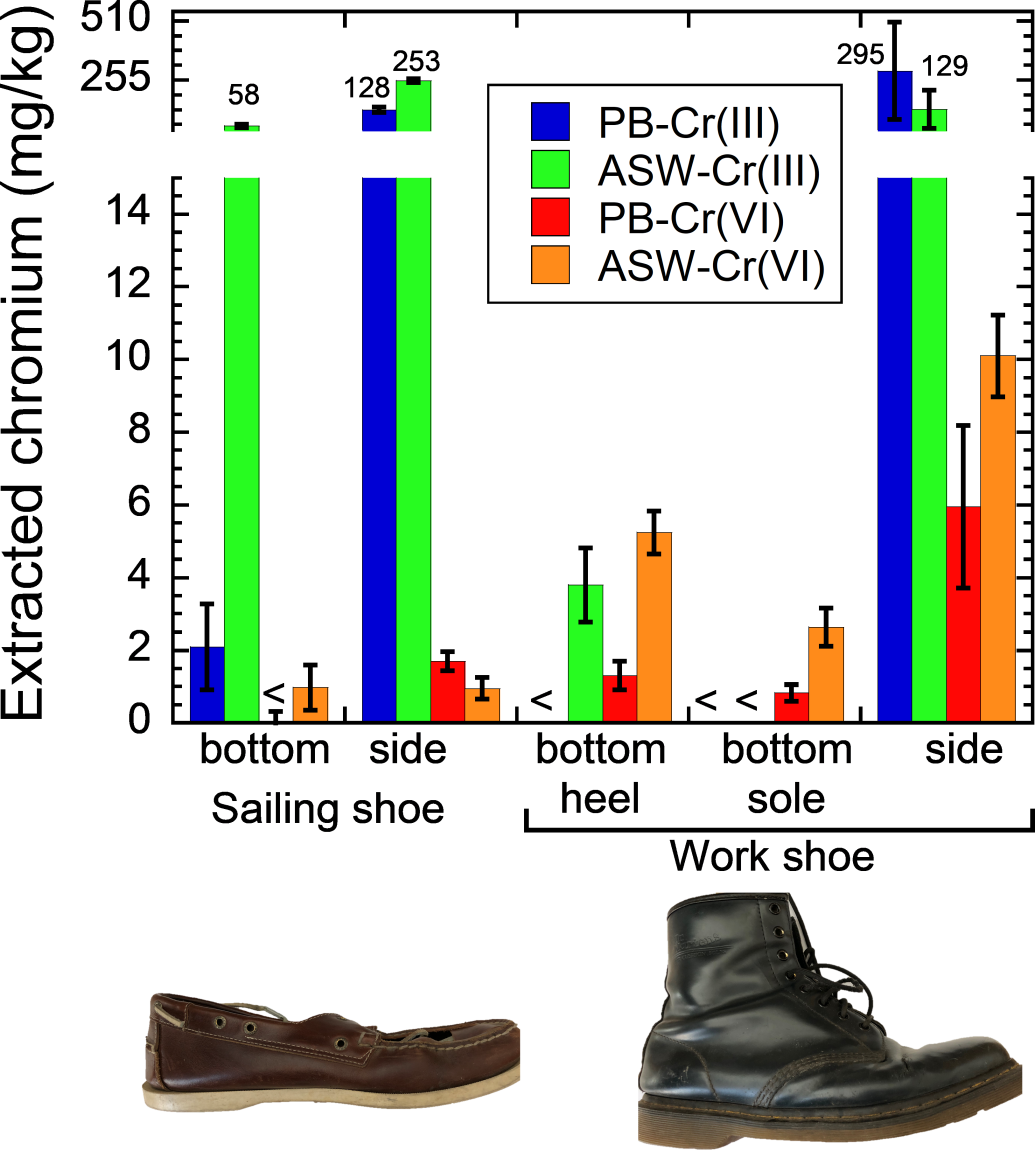
Extracted trivalent chromium (Cr(III)) and hexavalent chromium (Cr(VI)) from different parts of the sailing shoe and work shoe (shown in the bottom) in phosphate buffer (PB) after 3 hours (25°C) and artificial sweat (ASW) after 24 hours (30°C). The error bars show the standard deviation of three different leather pieces. Negative control is subtracted in all cases. <, below limit of detection

Cobalt was not detectable in any of the leather piece extractions. Extracted trivalent and hexavalent chromium are shown in Figure 1. The restriction limit of 3 mg/kg hexavalent chromium extracted in phosphate buffer was exceeded for leather of the side of the work shoe (10 mg/kg). For the bottom of the shoes, extracted trivalent chromium was significantly lower as compared to the sides of the shoes.

## DISCUSSION

A clear relevance of chromium allergy was found in this case of contact dermatitis due to chromium release from tanned leather, whereas the relevance of contact allergy to cobalt has not been revealed in our chemical analysis. Cobalt release from leather is known to be of potential relevance in contact allergy to cobalt^8^ but might have been nondetectable due to the age of the shoes. The release of hexavalent chromium was detectable in artificial sweat and even higher than in phosphate buffer in the case of the work shoes. This finding is most probably related to the age of the shoes. Previous studies on new leather have shown that released reducing agents efficiently reduce hexavalent to trivalent chromium in the more acidic artificial sweat.^9^ The detection of hexavalent chromium in artificial sweat and the relatively higher fraction of hexavalent to trivalent chromium in the bottom of the shoes are hence in line with our previous laboratory findings showing that the fraction of released hexavalent chromium as compared to trivalent chromium increases with leather age/use.^6^ In agreement with previous results for a number of international leather samples,^10^ there was no correlation found between released trivalent and hexavalent chromium. For example, the sailing shoe would not have been restricted according to registration, evaluation, authorisation and restriction of chemical substances (REACH),^3^ since the released amount of hexavalent chromium into phosphate buffer was less than 3 mg/kg. It released, however, up to 253 mg/kg trivalent chromium, which can also elicit contact dermatitis in response to chromium.^5^

The only readily available spot test method for chromium release, the diphenylcarbazide test, has not yet gained a standard place in the office of every practicing dermatologist. Therefore, the determination of the relevance of chromium allergy when patch testing is mostly based on guessing. Furthermore, the diphenylcarbazide method has sensitivity limits, as illustrated in our case. In cases when hand and foot dermatitis is mainly unifactorial, for example, due to contact allergy to chromium, the patient and the dermatologist will discover clear relevance when the patient changes to nonleather products. However, unifactorial cases are rare. The avoidance of chromium‐releasing items will not always lead to such an immediate relief of symptoms, which will make it difficult to establish relevance. Moreover, without chemical analysis, identification of alternative shoes and gloves for future use is highly unreliable. When purchasing new leather items, the customer can only rely on information a shop gives to the patient on chromium content in leather products, which is sometimes erroneous. Without any requirement on the declaration, or legally set limits on the release, of trivalent chromium in leather, no new leather items can reliably be purchased for patients with strong chromium allergy. To the best of our knowledge, even the most restrictive standard today, the OEKO‐TEX standard for leather, only limits trivalent chromium to <200 mg/kg in artificial sweat (except for baby shoes).^11^

In conclusion, we strongly recommend the use of analytical methods in the investigation of relevance at cases of chromium allergy when available. Moreover, we strongly plead for the consideration of extractable trivalent chromium in future legislative actions and declaration requirements. The presented case also underscores the importance of leather age for the release of hexavalent chromium.

## CONFLICTS OF INTERESTS

The authors declare no conflicts of interests.
